# Development and Application of an ELISA for the Detection of Porcine Deltacoronavirus IgG Antibodies

**DOI:** 10.1371/journal.pone.0124363

**Published:** 2015-04-16

**Authors:** Anil Thachil, Priscilla F. Gerber, Chao-Ting Xiao, Yao-Wei Huang, Tanja Opriessnig

**Affiliations:** 1 Department of Veterinary Diagnostic and Production Animal Medicine, Iowa State University, Ames, Iowa, United States of America; 2 The Roslin Institute and The Royal (Dick) School of Veterinary Studies, University of Edinburgh, Midlothian, United Kingdom; 3 Institute of Preventive Veterinary Medicine, College of Animal Sciences, Zhejiang University, Hangzhou, China; Ella Foundation, INDIA

## Abstract

Porcine deltacoronavirus (PDCoV), also known as porcine coronavirus HKU15, was first detected in North America in early 2014 and associated with enteric disease in pigs, resulting in an urgent need to further investigate the ecology of this virus. While assays detecting nucleic acids were implemented quickly, assays to detect anti-PDCoV antibodies have not been available. In this study, an indirect anti-PDCoV IgG enzyme-linked immunosorbent assay (ELISA) based on the putative S1 portion of the spike protein was developed and utilized to determine the prevalence of anti-PDCoV IgG in U.S. pigs. The diagnostic sensitivity of the PDCoV ELISA was 91% with a diagnostic specificity of 95%. A total of 968 serum samples were tested including samples with confirmed infection with PDCoV, porcine epidemic diarrhea virus (PEDV), transmissible gastroenteritis virus or porcine respiratory coronavirus. There was no cross-reactivity with any of the other coronaviruses. Among 355 arbitrarily selected serum samples collected in 2014 and originating from 51 farms across 18 U.S. states, anti-PDCoV IgG antibodies were detected in 8.7% of the samples and in 25.5% of the farms whereas anti-PEDV IgG was detected in 22.8% of the samples and in 54.9% of the farms. In addition, anti-PDCoV IgG antibodies were detected in archived samples collected in 2010, perhaps indicating an earlier undetected introduction into the U.S. pig population. Overall, the obtained data suggest that PDCoV seroprevalence in U.S. pigs is lower compared to PEDV and PDCoV may have been introduced to the U.S. prior to PEDV.

## Introduction

Porcine deltacoronavirus (PDCoV) was first identified in a rectal swab collected in 2009 from a pig in Hong Kong, China, and is related to avian and Asian leopard deltacoronaviruses identified in apparently healthy wild animals [1–3]. Recently, PDCoV has been described in association with diarrhea in pigs across all production stages in North America [4–6]. Specifically, in February 2014, the Ohio Department of Agriculture issued a press release indicating that PDCoV was detected in swine feces from five separate pig farms in Ohio, with clinical signs of watery diarrhea in sows and enteric disease associated with increased mortality in piglets [4]. The presence of transmissible gastroenteritis virus (TGEV) was ruled out but a portion of the samples were also positive for porcine epidemic diarrhea virus (PEDV) [4]. In response to the impact of the newly emerging porcine coronaviruses PEDV [7,8] and PDCoV [4] in the U.S. pig population, the USDA issued a federal order requiring reporting of all novel cases associated with all porcine enteric coronaviruses effective June 5, 2014 [9]. In contrast to PEDV [10–12], the epidemiology, viral pathogenesis and clinical symptoms associated with PDCoV infection are still largely unknown. Nevertheless, preliminary results obtained after experimental infection of gnotobiotic pigs with this virus indicate severe clinical signs (diarrhea, vomiting, dehydration) and microscopic lesions consistent with coronavirus infection in the small intestines [13]. However, serological assays to undertake field based epidemiological studies are not available to date. Sequencing of the complete genome of U.S. PDCoV strains from different states indicates a nucleotide identity of approximately 99% when compared to the PDCoV sequence initially reported in China [6,14,15].

While there is limited information available regarding coronaviruses in the genus *Deltacoronavirus*, other porcine coronaviruses belonging to the genus *Alphacoronavirus* such as PEDV, TGEV and the porcine respiratory coronavirus (PRCV) are well characterized [1,16]. In general, coronaviruses are enveloped, single-stranded RNA viruses and contain four major structural proteins: spike (S), envelope (E), membrane (M) and nucleocapsid (N) [17,18]. The S protein region of coronaviruses contains major neutralizing epitopes and also stimulates induction of neutralizing antibodies in the host [17,19,20]. Previously, the entire S protein or portions thereof have been successfully utilized for PEDV and TGEV ELISA development [21–24].

Diagnostic tools to demonstrate PDCoV infection have been largely limited to detection of nucleic acids by reverse transcriptase (RT) PCRs. To the author’s knowledge, serology assays to evaluate the humoral immune response against PDCoV have not been described to date. The objective of this study was to develop and utilize an anti-PDCoV IgG ELISA to investigate the prevalence rates of PDCoV in the U.S. pig population between 2006 and 2014, and provide a tool for advancing understanding of the epidemiology of PDCoV. Additionally, the prevalence rate of anti-PEDV IgG antibody was investigated in samples obtained in 2014.

## Materials and Methods

### Ethics statement

All samples utilized were arbitrarily selected and originated from pig case submissions to the Iowa State University Veterinary Diagnostic Laboratory (ISU-VDL) for diagnostic work-up. The sample collection and submission was unrelated to and not part of this study. The protocol for this study was approved by the Iowa State University Institutional Biosafety Committee.

### Samples

A total of 968 serum samples and 52 pooled porcine plasma samples were used in this study. All the samples were stored at −20°C until tested. Based on sample type and PDCoV exposure status, the samples were divided into three groups.

#### Serum samples from sows with known PDCoV exposure status (n = 210)

A total of 180 serum samples were collected from three U.S. farms (Farms A, B and C) with confirmed PDCoV exposure between May and August 2014, and 30 samples were collected from confirmed PDCoV negative Farm D in July 2013. Specifically, 60 serum samples were collected from 30 sows acutely affected by diarrhea and again four weeks later on Farm A. Another 120 serum samples were collected from Farms B and C in which pigs had been diagnosed with PDCoV at least two months prior to the serum collection (*n* = 60/farm). The last 30 samples came from PDCoV negative Farm D. The PDCoV infection status was determined based on real-time RT-PCR for PDCoV on fecal samples. PEDV, PRCV and TGEV RNA were not detected in the feces. All RT-PCRs were performed according to routinely performed standard protocols at the ISU-VDL. Farm A samples collected at the first time point and samples from the negative farm D were classified as negative (*n* = 60). Farm A samples collected at the second time point and samples from the two positive Farms B and C were classified as positive (*n* = 150). For the paired sample set from Farm A, PDCoV seroconversion was defined as a four-fold increase in the antibody titers when comparing the first and second sample collection points.

#### Serum samples collected from pigs with unknown PDCoV exposure status (n = 758)

A total of 355 serum samples were arbitrarily selected during 2014 from U.S. pig farms stratified by geographic origin as part of unrelated diagnostic submissions to the ISU-VDL. Five serum samples were selected from each of 71 independent submission cases from 51 different farms (*n* = 5 to 25 samples per farm) located in 18 different states across the U.S. (Colorado, Iowa, Illinois, Indiana, Kansas, Kentucky, Michigan, Minnesota, Missouri, Montana, Nebraska, New Jersey, North Carolina, Ohio, Oklahoma, Pennsylvania, South Dakota, and Wisconsin). All 355 samples were tested for presence of anti-PDCoV IgG and for anti-PEDV IgG antibodies using an assay previously described [25].

Archived serum samples (*n* = 403) collected from 2006 to 2013 prior to initial recognition of PDCoV from 25 different farms from six different states in the U.S. (Iowa, Illinois, Nebraska, North Carolina, Missouri, Texas) [25–27] were also included and tested for presence of anti-PDCoV IgG.

#### Pooled plasma samples collected from pigs with unknown PDCoV exposure

A total of 52 pooled liquid porcine plasma samples collected in 2010 as part of a previous study [28] were selected for testing. Each porcine plasma sample consisted of pooled plasma originating from exsanguination of approximately 10,000 pigs slaughtered on the same day. The samples were collected from 14 federally inspected abattoirs located in the eastern part or in the Midwestern U.S. [28]. Based on real-time RT-PCR testing, all samples obtained in 2010 were negative for PDCoV RNA.

### PDCoV S1 ELISA development

#### Protein production

The region encoding the putative S1 domain (amino acids 1–573) of the PDCoV strain PDCoV-IA2014-1 (GenBank accession number KM613173) was 3’ terminally fused with a thrombin cleavage sequence followed by a human IgG Fc domain, which was subsequently cloned into a eukaryotic expression vector as previously described [25]. The S1-Fc fusion proteins were expressed by transfection of HEK-293T cells, purified using protein A column purification, cleaved with thrombin to remove the Fc tag, and treated for endotoxin removal.

#### Assay optimization

The optimal antigen concentration and the serum dilution for the S1-PDCoV ELISA were determined using a checkerboard titration. Microtiter plates (Nunc; Thermo Fisher Scientific, Agawam, MA, USA) were coated with the S1 polypeptide diluted in coating buffer (50 mM carbonate buffer, pH 9.6) at a concentration of 0.95 ng per well and incubated overnight at 4°C. After three washes with PBS containing 0.05% Tween 20 (PBST), the plates were blocked with 1% bovine serum albumin (Jackson ImmunoResearch, West Grove, PA, USA) for 2 h at 22°C and then incubated with the serum or plasma samples diluted 1:100 in PBS containing 10% goat serum (Gibco; Life Technologies, Grand Island, NY, USA) for 30 min at 37°C. After a washing step, a 1:10,000 diluted peroxidase-conjugated goat anti-swine IgG (Jackson ImmunoResearch) was added and incubated at 37°C for 30 min. The peroxidase reaction was visualized by using tetramethylbenzidine-hydrogen peroxide solution as the substrate (KPL, Gaithersburg, MD, USA) for 10 min at room temperature and stopped by adding 50 μL of 2 M sulfuric acid to each well. Optical densities (OD) were measured at 450 nm using an ELISA plate reader (BioTek, Winooski, VT, USA). Serum dilutions that gave the greatest ratio between the positive and the negative sample (P/N) were selected as controls for subsequent runs and positive, negative and blank (sterile water) samples were tested in duplicate and included on each plate.

#### Assay specificity and cut-off value determination

The specificity of the PDCoV S1 ELISA was evaluated by using serum samples single-positive and with high antibody levels against TGEV (*n* = 30), PRCV (*n* = 30) or PEDV (*n* = 30) which were obtained through the ISU-VDL. The cut-off was calculated by receiver operator characteristic (ROC) analysis for maximum diagnostic sensitivity and specificity using samples classified as PDCoV positive (*n* = 150) or negative (*n* = 60 samples). The cutoff value was selected to maximize sensitivity and specificity while minimizing the sums of false negative and false positive results. The obtained value was further evaluated using the cumulative data from all other samples. The ROC was defined to determine the cut-off of the PDCoV S1 ELISA using MedCalc for Windows, version 13.3.0.0. (MedCalc Software, Ostend, Belgium).

#### Assay reproducibility

The reproducibility of the PDCoV S1 ELISA was evaluated by utilizing eight serum samples with different antibody titers. The coefficient of variation (CV) was used to evaluate the intra- and inter-assay variation. Each sample was tested on each of three plates on different occasions to determine the inter-assay CV, and three replicates within the same plate were used to calculate the intra-assay CV.

## Results

### PDCoV S1 ELISA development

**Fig 1:**
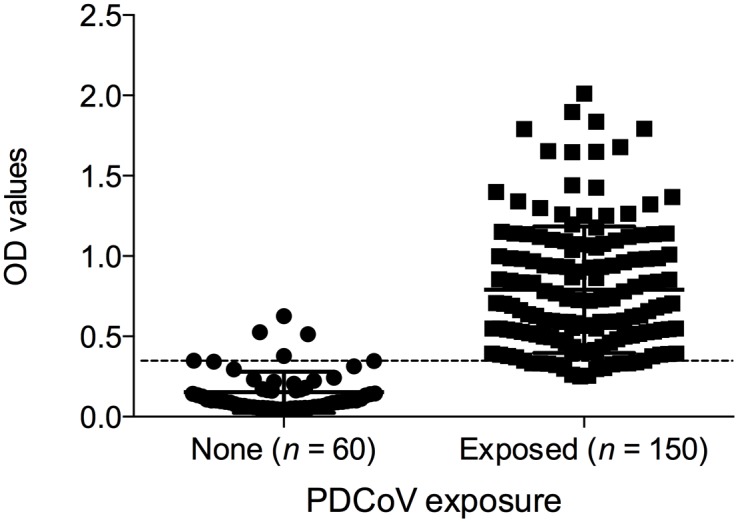
Distribution of serum anti-PDCoV IgG antibodies obtained from farms with known PDCoV exposure. Serum samples were classified as negative or positive based on viral RNA detection on fecal samples at the farm. Data presented as ELISA OD values ± SEM. The assay cut-off (OD value of 0.34) is indicated by the dashed line.

The ROC analysis based on 210 serum samples with known PDCoV exposure was used for the cut-off determination (Fig 1). The optimal cut-off for the PDCoV S1 ELISA was a 1:100 diluted sample OD value of 0.34 for which the sensitivity and specificity values were higher than 90%. Sensitivity was 90.6% and the specificity was 94.8%. The diagnostic accuracy of PDCoV S1 ELISA was considered to be high as the area under the curve (AUC) index was 0.98 with a standard error of 0.01. Inter and intra-coefficient of variation (CV) of eight control sera tested with PDCoV S1 ELISA was less than 10%. The intra-assay CV ranged from 2.6% to 4.2% while the inter-assay CV ranged from 5.2% to 9.4%, indicating that the results were reproducible. The assay specificity was determined by using samples positive for antibodies to TGEV, PRCV or PEDV. The obtained PDCoV S1 ELISA OD values ranged from 0.04 to 0.33 (average ± SD, 0.12 ± 0.06), indicating a lack of cross-reaction with other porcine coronaviruses. Among the 90 samples positive for antibodies against TGEV, PRCV or PEDV, only one had an OD value higher than 0.3 for PDCoV. Specifically, PDCoV OD values ranged from 0.04 to 0.33 (average ± SD, 0.12 ± 0.08) for TGEV; 0.05 to 0.291 (average ± SD, 0.10 ± 0.06) for PRCV; and 0.07 to 0.244 (average ± SD, 0.13 ± 0.05) for PEDV.

### Presence of anti-PDCoV IgG antibodies in pig serum during an acute outbreak and four weeks later

**Fig 2:**
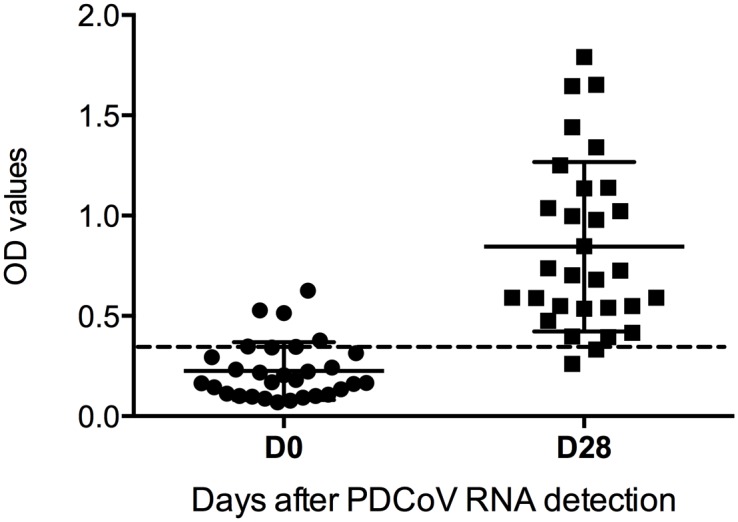
Distribution of serum anti-PDCoV IgG antibodies during an acute outbreak and four weeks later. An acute outbreak was defined as presence of clinical disease and demonstration of PDCoV RNA in feces. Data presented as ELISA OD values ± SEM. The assay cut-off (OD value of 0.34) is indicated by the dashed line.

Anti-PDCoV IgG antibody positive serum samples were detected in 7 (23.3%) of the 30 sows acutely affected by diarrhea and in 28 (93.3%) samples four weeks after PDCoV RT-PCR diagnosis (Fig 2). Two sows were seronegative at both collection times. Seroconversion, characterized by at least a four-fold increase in the OD values was detected in 16 (53.3%) sows. Among the seropositive sows identified during the first collection, 1/7 showed a seroconversion (4.4-fold increase in the OD values); 3/7 had at least a 2-fold increase in the OD values (average ± SD 2.46 ± 0.74); and 3/7 had OD value increments lower than 1.5-fold.

### Presence of anti-PDCoV and -PEDV IgG antibodies in pig serum samples with unknown PDCoV exposure collected during 2014

Anti-PDCoV and -PEDV IgG antibody prevalence rates in 355 serum samples collected during 2014 are summarized in Table 1. Thirty-one serum samples (8.7%) were anti-PDCoV IgG antibody positive which were identified in 13/51 (25.5%) farms. Positive detection rates in individual PDCoV farms ranged from 20 to 100%. Anti-PEDV IgG antibodies were detected in 81/355 (22.81%) serum samples and in 28/51 (54.9%) of the investigated farms. Concurrent detection of anti-PEDV and anti- PDCoV IgG antibodies occurred in 8/51 farms (15.7%) and 23/355 (6.5%) serum samples.

**Table 1:** Detection rate of anti-PDCoV and anti-PEDV IgG antibodies in pig sera samples collected during 2014.

State	Number of positive samples/number samples tested	
	(Number of positive farms/number of farms tested)	
	PDCoV	PEDV
Colorado	0/5 (0/1)	3/5 (1/1)
Iowa	12/145 (7/20)	21/145 (11/20)
Illinois	1/15 (1/3)	4/15 (2/3)
Indiana	3/20 (1/4)	7/20 (2/4)
Kansas	0/5 (1/1)	0/5 (1/1)
Kentucky	0/5 (0/1)	0/5 (0/1)
Michigan	0/5 (0/1)	0/5 (0/1)
Minnesota	0/5 (0/1)	0/5 (0/1)
Missouri	3/20 (1/3)	20/20 (3/3)
Montana	0/5 (0/1)	0/5 (0/1)
North Carolina	0/30 (0/2)	1/30 (1/2)
Nebraska	8/10 (2/2)	7/10 (2/2)
New Jersey	0/5 (0/1)	0/5 (0/1)
Ohio	0/5 (0/1)	0/5 (0/1)
Oklahoma	0/35 (0/4)	15/35 (2/4)
Pennsylvania	4/20 (1/3)	3/20 (1/3)
South Dakota	0/5 (0/1)	0/5 (0/1)
Wisconsin	0/5 (0/1)	0/5 (0/1)
**Total**	**31/355 (13/51)**	**81/355 (28/51)**

All serum samples were obtained from commercial pig farms in 18 different states across the U.S.A.

### Presence of anti-PDCoV IgG antibodies in pig serum and plasma samples with unknown PDCoV exposure collected prior to 2014

Among the 403 archived serum samples collected between 2006 and 2013, 44 (10.9%) serum samples were found to be positive for anti-PDCoV IgG antibodies by the S1 ELISA (Table 2). The majority of positive samples were collected in 2013 (40/44, 90.9%). On positive farms, 20 to 60% of the serum samples were found to be positive. Interestingly, four samples were collected in 2010; specifically, three serum samples originated on a farm in Illinois and one serum sample was from a farm in Iowa. The OD values on these samples were 0.38, 0.39, 0.42, and 1.27. Due to the limited availability of retrospective sera and to further confirm this finding, 52 porcine pooled plasma samples from 2010 were also tested. Two out of 52 (3.8%) pooled plasma samples were positive for anti-PDCoV IgG antibodies; the OD values were 0.34 and 0.38 whereas the OD values of negative samples ranged from 0.05 to 0.15 (average ± SD, 0.08 ± 0.01).

**Table 2:** Detection rate of anti-PDCoV IgG antibodies from 2006 to 2014 in the U.S.A.

Year	PDCoV positive samples/number of samples tested
	(PDCoV positive farms/number of farms tested)
2006	0/19 (0/1)
2007	0/16 (0/1)
2010	4/58 (2/4)
2011	0/9 (0/2)
2012	0/91 (0/9)
2013	40/210 (6/10)
2014	31/355 (13/51)
**Total**	**75/758 (21/78)**

All serum samples were obtained from commercial pig farms.

## Discussion

PDCoV was initially discovered in U.S. pigs in 2014 [4] leading to many questions on basic infection dynamics and time of introduction of this emergent pig virus. The objectives of this study were to develop a serological assay to evaluate the 2014 prevalence rates of PDCoV in U.S. pigs and to determine evidence for PDCoV infection in previous years.

In order to accomplish this goal, a recombinant PDCoV S1 polypeptide-based ELISA was developed and the S1 subunit was selected as coating antigen. The amino acid identity of the PDCoV S1 subunit and the corresponding PEDV counterpart (IA1 strain, GenBank accession number KF468753) is 20.2%, for TGEV (Purdue strain, GenBank accession number AJ271965) it is 20.7% and for PRCV (ISU-1 strain, GenBank accession number DQ811787) it is 22.1% [5]. Therefore, the PDCoV S1 subunit used in the present study was unlikely to cross-react with PEDV, PRCV or TGEV and as expected, cross-reactivity was not observed. Furthermore, *in silico* prediction of the PDCoV epitopes was performed and results were compared with the alphacoronavirus PEDV, TGEV and PRCV. As expected, the S1 protein contained the majority of the mapped epitopes within the coronavirus proteins known to elicit humoral response (E, M, N and S). In addition, a comparison of the amino acid identity between the expressed PDCoV S1 antigen for the developed ELISA and the other 19 PDCoV sequences in the GenBank indicated a similarity higher than 99% for the S1 polypeptide, which suggests that this segment is conserved enough to detect humoral responses directed to it.

As experimentally generated samples or a gold standard test for PDCoV antibody detection were not available at the time the study was conducted, field samples with known PDCoV exposure and from presumed negative pigs were utilized to gain insights on basic PDCoV seroconversion. Anti-PDCoV antibodies and seroconversion were detected within four weeks of initial observation of clinical disease and detection of PDCoV RNA in fecal samples. Ideally, the cut-off value should be derived by testing a panel of samples obtained from reference animals with known history and infection status relative to the disease [29]. Due to difficulties in classifying pigs from farms as true negative, i.e. no previous exposure to PDCoV which would have required to obtain fecal samples over-time from an adequate number of pigs on the farm, samples from the start of perceived PDCoV outbreaks (PDCoV real-time RT-PCR positive pigs) were considered to be seronegative for test development purposes. Although this strategy was sufficient to confirm diagnosis of acute infection as seroconversion was detected in paired samples from PDCoV outbreaks, the lack of a second serological assay and known negative samples did not permit a precise estimation of the diagnostic sensitivity and specificity of the test. Selecting a single arbitrary cut-off value entirely on the basis of field samples and association with clinical history and RT-PCR results could result in a loss of sensitivity and/or specificity. To further address this, the established cut-off value of 0.34 was evaluated using the cumulative data of 573 samples originating from seronegative farms (classified as negative due to absence of any PDCoV ELISA positive pig among the pigs tested) by calculating the average OD (data not shown). It was determined that the adoption of this method obtained lower cut-off value (0.24; OD average ± 3 × SD; average ± SD 0.09 ± 0.05), and provided a greater sensitivity. Therefore, samples with an OD value between 0.24 and 0.34 should perhaps be considered inconclusive. The lack of a gold standard for PDCoV antibody detection further contributes to the problem in establishing an appropriated cut-off and accurately measure the sensitivity and specificity of the ELISA developed herein.

Based on cumulative data from the National Animal Health Laboratory Network (NAHLN) laboratories through 17 Sep 2014, 6.6% (382) of 5827 cases obtained from 17 of 31 states were positive for PDCoV RNA [30]. In the present study, 8.7% (31/ 355) of the serum samples collected from 7/18 states were positive for anti-PDCoV IgG which is in agreement with the Animal and Public Health Information System (APHIS) surveillance data and PDCoV RNA detection by RT-PCR. In contrast, the percentage of PEDV RNA positive cases was 26.0% (8386/32211) obtained from a total of 31/42 U.S. states according to the most recent NAHLN survey [30]. In further agreement, in this study, 22.8% (81/355) of the samples arbitrarily selected in 2014 were positive for PEDV IgG.

It has been determined previously that co-infections of PDCoV with other enteric viruses are common [9]. In the present investigation, 38% of PEDV ELISA positive pigs were also positive for anti-PDCoV antibodies (data not shown). This is in agreement with previous reports which found that 78% of PDCoV RNA positive samples were either positive for PEDV RNA, rotavirus group A RNA, rotavirus group B RNA, or rotavirus group C RNA and 33% were coinfected with PEDV as determined by RT-PCR [14]. Experimental trials are needed to better understand the comparative virulence of PDCoV to other enteric pathogens.

PDCoV was first identified in U.S. pigs in 2014 [4] and retrospective studies targeting detection of RNA have been able to identify the virus in late 2013 [30]. Access to archived serum samples can be limited and to overcome this issue, archived porcine plasma samples were also used in this study. While the PEDV assay was not validated on plasma, evidence in the literature suggests that the degree of antibody detection in serum and plasma is essentially identical [31–34]. Overall, the obtained data suggest that PDCoV has been circulating in the North American pig population prior to 2013 without being recognized. Specifically, antibodies to PDCoV were detected in archived serum and plasma samples from 2010, whereas anti-PDCoV IgG antibodies were not detected in serum samples collected in 2011 or 2012. This could be due to the limited number of available retrospective serum samples in combination with an overall low PDCoV seroprevalence. Alternatively, the positive samples collected in 2010 could be false positive; however, at least one sample presented a high OD value. Upon availability of another PDCoV serological test in the future these results will need to be confirmed.

In conclusion, as already indicated by PDCoV RT-PCR surveillance results, the obtained anti-PDCoV IgG prevalence data further confirm an overall low prevalence rate of PDCoV infection in the U.S. pig population.
